# Striatal dopamine D_2_/D_3_ receptor binding in pathological gambling is correlated with mood-related impulsivity

**DOI:** 10.1016/j.neuroimage.2012.06.067

**Published:** 2012-10-15

**Authors:** Luke Clark, Paul R. Stokes, Kit Wu, Rosanna Michalczuk, Aaf Benecke, Ben J. Watson, Alice Egerton, Paola Piccini, David J. Nutt, Henrietta Bowden-Jones, Anne R. Lingford-Hughes

**Affiliations:** a Department of Experimental Psychology, University of Cambridge, Cambridge, UK; b Centre for Neuropsychopharmacology, Division of Brain Sciences, Department of Medicine, Imperial College London, UK; c Centre for Neuroinflammation and Neurodegeneration, Division of Brain Sciences, Department of Medicine, Imperial College London, UK; d Psychopharmacology Unit, University of Bristol, UK; e Department of Psychosis Studies, Institute of Psychiatry, King's College London, UK; f National Problem Gambling Clinic, Central and North West London NHS Foundation Trust, UK; g Centre for Mental Health, Division of Brain Sciences, Department of Medicine, Imperial College London, UK

**Keywords:** Gambling, Impulsivity, Dopamine, Neuroimaging, Addiction, Striatum

## Abstract

Pathological gambling (PG) is a behavioural addiction associated with elevated impulsivity and suspected dopamine dysregulation. Reduced striatal dopamine D_2_/D_3_ receptor availability has been reported in drug addiction, and may constitute a premorbid vulnerability marker for addictive disorders. The aim of the present study was to assess striatal dopamine D_2_/D_3_ receptor availability in PG, and its association with trait impulsivity. Males with PG (n = 9) and male healthy controls (n = 9) underwent [11C]-raclopride positron emission tomography imaging and completed the UPPS-P impulsivity scale. There was no significant difference between groups in striatal dopamine D_2_/D_3_ receptor availability, in contrast to previous reports in drug addiction. However, mood-related impulsivity (‘Urgency’) was negatively correlated with [11C]-raclopride binding potentials in the PG group. The absence of a group difference in striatal dopamine binding implies a distinction between behavioural addictions and drug addictions. Nevertheless, our data indicate heterogeneity in dopamine receptor availability in disordered gambling, such that individuals with high mood-related impulsivity may show differential benefits from dopamine-based medications.

## Highlights

► Assessed 11C-raclopride binding in pathological gambling, a putative behavioral addiction. ► No group difference in striatal dopamine binding from healthy controls. ► Dopamine binding negatively correlated with mood-related impulsivity (‘Urgency’).

## Introduction

Pathological Gambling (PG) is a DSM-IV impulse control disorder with substantial clinical and aetiological overlap with drug addiction, prompting a re-conceptualisation of PG as a ‘behavioural addiction’ (Bowden-Jones and Clark, 2011; Frascella et al., 2010). Neurobiological models of drug addiction emphasise the dysregulation of dopamine: many drugs of abuse stimulate dopamine neurotransmission (Di Chiara and Imperato, 1988), and reductions in dopamine D_2_/D_3_ receptor availability have been described in patients dependent upon a variety of abused drugs (Fehr et al., 2008; Heinz et al., 2004; Martinez et al., 2004; Volkow et al., 1997, 2001). It is unclear whether these alterations reflect a consequence of long-term drug use, or a pre-existing vulnerability to addiction. Consistent with a vulnerability marker, ‘drug-liking’ is associated with low D_2_/D_3_ receptor availability (Volkow et al., 1999), and a rodent strain inbred to be behaviourally impulsive displayed rapid acquisition of cocaine self-administration and reduced striatal dopamine D_2_/D_3_ receptor availability prior to drug exposure (Dalley et al., 2007). As a form of addiction with presumably negligible toxicity, studies of PG may enable further study of vulnerability models in humans, and help arbitrate issues of cause and consequence (Verdejo-Garcia et al., 2008). Indeed, there are a number of indications of dopamine dysregulation in PG. Peripheral dopamine markers in cerebrospinal fluid are dysregulated in problem gamblers (Bergh et al., 1997; Meyer et al., 2004), as are fMRI responses in dopamine-rich circuitry during performance on gambling tasks (Chase and Clark, 2010; Reuter et al., 2005), although the direction of effect is inconsistent. In addition, dopamine-agonist medications for Parkinson's Disease appear capable of triggering disordered gambling as a side-effect (Voon et al., 2009).

Positron Emission Tomography (PET) imaging with [11C]-raclopride provides a means of quantifying striatal dopamine transmission in the living human brain. [11C]-raclopride has recently been used in four PET studies scanning participants with disordered gambling in dynamic (i.e. task-related) designs (Joutsa et al., 2012; Linnet et al., 2011; O'Sullivan et al., 2011; Steeves et al., 2009). Two of these studies were in patients with Parkinson's Disease (O'Sullivan et al., 2011; Steeves et al., 2009), where it remains unclear how the range of associated impulse control disorders are functionally related to the primary neuropathology of the disease (Voon et al., 2009). The other two studies, in primary PG, both used complex decision-making / gambling tasks where the baseline scan involved a sensorimotor control task (Joutsa et al., 2012; Linnet et al., 2011). Only one study (Steeves et al., 2009) found evidence for reduced striatal dopamine D_2_/D_3_ receptor availability in the group with disordered gambling. The present study examined baseline striatal dopamine D_2_/D_3_ receptor availability in treatment-seeking patients with a primary diagnosis of PG, where we hypothesized a reduction in D_2_/D_3_ receptor availability based on prior studies in drug addiction.

We also sought to explore striatal D_2_/D_3_ receptor availability in relation to trait impulsivity. Elevated impulsivity is reliably observed across both drug addictions and PG (Verdejo-Garcia et al., 2008), and is also seen to prospectively predict the development of substance use and gambling problems (Slutske et al., 2005). We recently used the UPPS-P impulsivity scale (Cyders et al., 2007) to assess subfacets of the impulsivity construct in patients with PG attending the UK National Problem Gambling Clinic (Michalczuk et al., 2011). Significant differences were observed between the PG group and healthy controls on several of the UPPS-P subscales including Urgency – the tendency to be impulsive during negative or positive mood states (‘rash impulsivity’) – and aspects of ‘narrow’ impulsivity (Lack of Planning and Lack of Perseverance). However, the effect sizes for the Urgency differences were markedly higher than for the narrow impulsivity facets, prompting the conclusion that mood-related impulsivity is especially relevant in the context of disordered gambling (Michalczuk et al., 2011). In light of these observations, our individual differences analyses focussed *a priori* on the two Urgency subscales (Negative Urgency and Positive Urgency) as predictors.

## Methods and materials

### Participants

Nine male volunteers with PG (mean age 35.3 years, sd 9.0, range 25–49) were compared against nine male healthy controls (mean age 37.2, sd 5.6, range 30–46). A tenth PG subject was recruited but not available for analysis due to radiochemistry failure. Control volunteer baseline scans were identified from two previous studies (Egerton et al., 2010; Stokes et al., 2010) using a normative database of raclopride scans held at the MRC Clinical Sciences Centre, and did not differ from the PG group in age (t_16_ = 0.53, p = .602). The PG participants were educated to at least high-school level with IQ estimates in the healthy range (Wechsler Adult Scale for Intelligence: mean 116, sd 10.8; National Adult Reading Test: mean 117, sd 5.7); past work indicates no consistent relationship between intelligence and dopamine binding levels. All volunteers provided written informed consent for the study, which was approved both by the Hammersmith Research Ethics Committee and the Administration of Radioactive Substances Advisory Committee, UK.

PG participants were recruited from the National Problem Gambling Clinic, Central North West London NHS Foundation Trust. Six volunteers were imaged shortly prior to, or during, a ten session course of cognitive-behavioural therapy and three had recently completed treatment. All nine volunteers had a recent history of active gambling. DSM-IV diagnosis of PG was confirmed with the Massachusetts Gambling Screen (MAGS; mean 9.8, sd 2.2, range 5–12) (Shaffer et al., 1994), administered by an assistant psychologist at the time of treatment initiation. The diagnosis was corroborated with the Problem Gambling Severity Index (Ferris and Wynne, 2001), a self-report scale also given at the beginning of treatment (mean 18.4, sd 5.7, range 8–24; a score of 8 or above indicates problem gambling). The delay between the clinical evaluation and the PET scan was 2–8 months in 8/9 participants, and 23 months in one PG who was scanned post-treatment. Psychiatric co-morbidities were assessed in the PG participants by a semi-structured interview using the ICD-10, in conjunction with the computerised version of the Mini International Neuropsychiatric Interview (e-MINI v2.0; Medical Outcome Systems, Jacksonville, Florida) (Sheehan et al., 1998). Two volunteers had a previous history of major depressive disorder, and one volunteer met criteria for current and lifetime major depressive disorder. One volunteer met criteria for previous alcohol dependence, and a second met criteria for previous cannabis dependence. Four volunteers were current cigarette smokers at the time of recruitment to the study (Fagerstrom Nicotine Dependence Scale scores from 6 to 12). Exclusion criteria for the PG group were: history of neurological illness, previous psychiatric admission, current pharmacotherapy and significant physical illness. As such, the detected psychiatric comorbidities were not of sufficient severity to require clinical intervention. All of the control participants had been previously assessed by a psychiatrist to exclude current or previous significant mental health problems, and substance dependence as defined by DSM-IV, serious physical illness, past neurological disorders or previous use of psychotropic medications.

Gambling activities were assessed in the PG participants using items 1–3 on the South Oaks Gambling Screen (Lesieur and Blume, 1987). Six participants considered electronic gaming machines (‘Fixed Odds Betting Terminals’) to represent their problematic form of gambling; the remaining three considered sports betting (on horses), internet poker / blackjack, and casino games (roulette) to be most problematic. On rating the largest amount of money gambled in a single day, five endorsed the £1,000-£10,000 bin, and four endorsed over £10,000. When questioned about gambling-related debts, one gambler refused to provide debt information, one gambler reported no debt due to use of personal savings, and current debt in the remaining seven ranged from £4000–£35,000 (mean £15,714).

Participants completed the UPPS-P Impulsive Behaviour Scale (Cyders et al., 2007), a 59-item self-report questionnaire with five subscales assessing Negative Urgency (e.g. “Sometimes when I feel bad, I can't seem to stop what I am doing even though it is making me feel worse”), Positive Urgency (e.g. “When overjoyed, I feel like I can't stop myself from going overboard”) , (lack of) Planning (e.g. “I usually make up my mind through careful reasoning” — negative loading), (lack of) Perseverance (e.g. “I finish what I start” — negative loading) and Sensation Seeking (e.g. “I would enjoy the sensation of skiing very fast down a high mountain slope”). We were unable to obtain UPPS-P data from one of the database control volunteers.

### Image acquisition and processing

All PET scans were acquired using an ECAT HR + 962 scanner (CTI/Seimens) with an axial field of view of 15.5 cm. [11C]-raclopride was administered as an intravenous bolus injection for PG volunteers, and for the control volunteers as an initial intravenous bolus followed by constant infusion, with an infusion length of 85 min for four scans from the Egerton et al. study (2010) and 100 min for five scans from the Stokes et al. study (2010). A 10 min transmission scan was performed prior to each emission scan to measure and correct for tissue attenuation. Dynamic emission scans were acquired in three dimensional mode using a standard acquisition protocol (20 time frames over 60 min for PG participants, 28 time frames over 85 min for the Egerton et al. (2010) scans and 38 frames over 100 minutes for the Stokes et al. (2010) scans). For the PG participants, their scan involved the presentation of neutral images including landscapes, household objects and random patterns, but with no motor requirement (participants were only scanned once).

All dynamic scans were corrected for head movement using frame by frame (FBF) realignment (Montgomery et al., 2006). This procedure was applied to all frames to generate an FBF-corrected dynamic image, which was then analysed using an automated region of interest (ROI) analysis, supplemented by a confirmatory voxelwise analysis.

### ROI analysis

Striatal and cerebellar ROIs were defined using an atlas comprised of the three functional subdivisions of the striatum; limbic, associative and sensorimotor striatum, and the cerebellum as reference region. The striatal subdivisions are anatomically analogous to the ventral striatum (limbic striatum), pre-commissural dorsal putamen, pre-commissural dorsal caudate and post-commissural dorsal caudate (associative striatum) and post-commissural putamen (sensorimotor striatum) (Martinez et al., 2003). An [11C]-raclopride template was spatially transformed to the individual PET space of each FBF-corrected add image (generated from each FBF-corrected dynamic image using in house software written in Matlab (version 5; The MathWorks, Inc, Natick, Mass)) within SPM5 (www.fil.ion.ucl.ac.uk/spm) and the resulting deformation matrix was then applied to the atlas. The deformed striatal atlas was used to sample counts from dynamic [11C]-raclopride images for the PG scans, and from a weighted steady state add image for control scans, using Analyze 8.0 software (www.analyzedirect.com). For PG scans, [11C]-raclopride BP_ND_ values, the ratio of specifically bound radioligand to that of the non-displaceable ligand in the cerebellar reference tissue (Innis et al., 2007), were calculated using a simplified reference tissue model with the cerebellum as a reference tissue using in house software written in Matlab. For control scans, [11C]-raclopride BP_ND_ values were calculated as the ratio of striatal counts to cerebellar counts, minus 1, over the steady state time period. The steady state time period for the bolus infusion scans was defined as commencing at 39 min post-injection and continuing until the end of the scan, based on estimates of the optimal timing for the establishment of the steady state (Watabe et al., 2000).

### Voxelwise analysis

For the PG volunteer scans, parametric [11C]-raclopride images were generated from individual dynamic images using a simplified reference tissue model with the cerebellum as a reference tissue using in house software written in Matlab. For control volunteer scans, parametric images were generated from individual weighted steady state add images using image algebra within SPM5 by dividing counts for each voxel with cerebellar counts and subtracting one. All parametric images were then normalised to an [11C]-raclopride PET template using the deformation matrix produced by the spatial transformation of individual add images to the template. Normalised parametric images were then smoothed within SPM5 using a 6 mm smoothing kernel.

### Statistical analysis

Group differences in impulsivity and regional BP_ND_ values were assessed using multivariate analysis of variance (MANOVA), implemented in SPSS 15 (SPSS, Chicago, Illinois). The relationships between Urgency and BP_ND_ values were assessed using partial correlation coefficients, controlling for age given the robust influence of age upon PET measures of D_2_/D_3_ receptor availability even within middle adulthood (Backman et al., 2000; Kim et al., 2011). A Bonferroni-corrected statistical threshold of p < .00625 was implemented for the correlational analyses, adjusting for the four striatal regions (overall, limbic, associative, sensorimotor) and two Urgency scales. For the voxelwise analysis, correlations between Urgency and [11C]-raclopride binding were examined using a multiple regression analysis within SPM5, restricted to the striatum, and again included volunteer age as a covariate. A corrected cluster level threshold of p < 0.05 with a cluster size of greater than ten voxels was used for statistical significance.

## Results

On the UPPS-P impulsivity scale, there was an overall main effect of group (Wilks’ lambda = 0.21, F(5,11) = 8.44, p = .002) with the PG group scoring significantly higher than healthy controls on the Negative Urgency (F_1,15_ = 43.0, p < .001), Positive Urgency (F_1,15_ = 17.4, p = .001), and (lack of) Planning (F_1,15_ = 4.95, p = .042) subscales. Consistent with our recent report in an extended group of PG recruited through the same clinic (Michalczuk et al., 2011), the effect sizes were greatest on the two Urgency subscales (see Table 1). There were no significant differences between groups on (lack of) Perseverance (F_1,15_ = 0.59, p = .455) and Sensation Seeking (F_1,15_ = 0.76, p = .398).

### ROI analysis

**Fig. 1:**
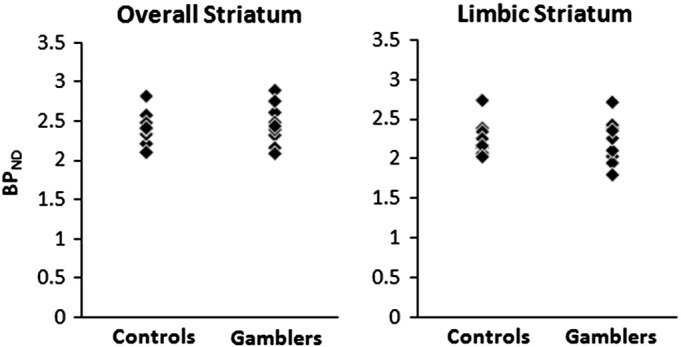
[11C]-raclopride binding potentials (BP_ND_) for the overall striatum region of interest (bilateral) and limbic subdivision, for individual cases with Pathological Gambling and healthy controls.

There was no overall group difference in [11C]-raclopride BP_ND_ values (Wilks' lambda = 0.59, F(4,13) = 2.22, p = .124), with no difference in the overall striatum (F_1,16_ = 0.22, p = .64) or in any of the three striatal subdivisions (limbic F_1,16_ = 0.02, p = 0.879; associative F_1,16_ = 0.54, p = 0.473; sensorimotor F_1,16_ = 0.05, p = .819) (see Fig. 1 and Table 1).

**Fig. 2:**
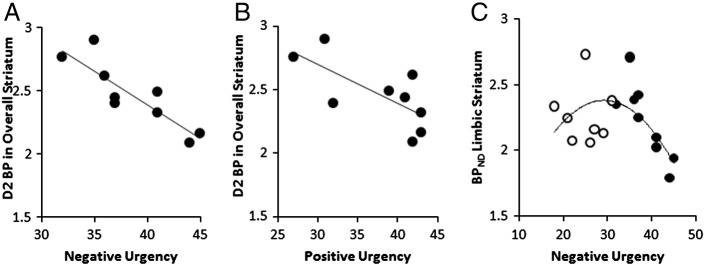
Correlations in Pathological Gamblers between [11C]-raclopride BP_ND_ in overall striatum and UPPS-P Negative Urgency (A) and Positive Urgency (B). C: Quadratic relationship between [11C]-raclopride BP_ND_ in limbic striatum and Negative Urgency in the pooled group of pathological gamblers (filled circles) and healthy controls (open circles).

Within the PG group, negative correlations (partialling for age) were observed between Negative Urgency and [11C]-raclopride BP_ND_ values in the overall striatum (Fig. 2A), and limbic and associative subdivisions of the striatum, each of which was significant at the Bonferroni-corrected level (see Table 2). Positive Urgency was significantly negatively correlated with BP_ND_ values in the overall striatum (Fig. 2B), and associative subdivision, at the corrected threshold. Negative Urgency and Positive Urgency were themselves moderately inter-related (57% shared variance in the PG, 81% shared variance in controls). The relationships between Urgency and BP_ND_ were not obviously explained the presence of four smokers in the PG group: the smokers (versus PG non-smokers) displayed negligible differences in Urgency scores (Negative Urgency mean = 39.0 vs 38.4; Positive Urgency mean = 38.8 vs 37.0) and BP_ND_ values (limbic striatum mean = 2.20 vs 2.24). Without age included as a partial variable, the BP_ND_ correlations remained significant for Negative Urgency (overall r_9_ = −.875, p = .002; limbic r_9_ = −.846, p = .004; associative r_9_ = −.868, p = .002) but the correlations against Positive Urgency did not attain significance at the corrected threshold (overall r_9_ = −.703, p = .035; associative r_9_ = −.738, p = .023). Scores on the Problem Gambling Severity Index (PGSI) were inversely correlated with BP_ND_ values in the associative striatum (partial rho = −.881, p = .004) but were non-significant without age included as a partial variable (r_9_ > − 0.45, p > 0.22). PGSI was highly correlated with Positive Urgency (r_9_ = .916, p < .001) but not significantly with Negative Urgency (r_9_ = .627, p = .071).

BP_ND_ values were not significantly correlated with the Urgency measures in the control group (r = − 0.36 to 0.31, p > 0.42). Indeed, for the relationship between Negative Urgency and BP_ND_ in the limbic striatum, a direct test of the difference between the correlation co-efficients confirmed a stronger relationship in the PG group compared to the controls (Fisher's r to z transformation; z = 2.03, p = .043), although equivalent tests in the overall striatum for Negative Urgency (z = 1.48, p = .139) and Positive Urgency (z = 0.97, p = .332) were not significant. Given the group increase in mood-related impulsivity in the PG group, we also ran a post-hoc analysis to test for a quadratic relationship between Urgency and [11C]-raclopride BP_ND_ in the pooled sample, in light of a recent report of an ‘inverted U’ relationship between ventral striatal raclopride binding and trait Sensation Seeking in healthy volunteers (Gjedde et al., 2010). In regressing [11C]-raclopride binding values in the limbic striatum (dependent variable) onto Negative Urgency (predictor variable), the overall model did not attain significance (F(2,14) = 3.65, p = .053) but there was a significant effect of the quadratic term (β = − 4.07, t = − 2.21, p = .045) (Fig. 2C). These quadratic effects were not observed for Positive Urgency in the limbic striatum (β = − 2.30, t = − 1.40, p = .183), or in the overall striatum (Negative Urgency: β = − 3.10, t = − 1.56, p = .141; Positive Urgency: β = − 1.75, t = − 1.06, p = .306). A direct attempt to replicate the quadratic effect for Sensation Seeking in the limbic striatum was also non-significant (β = 1.35, t = 0.44, p = .664).

### Voxelwise analysis

**Fig. 3:**
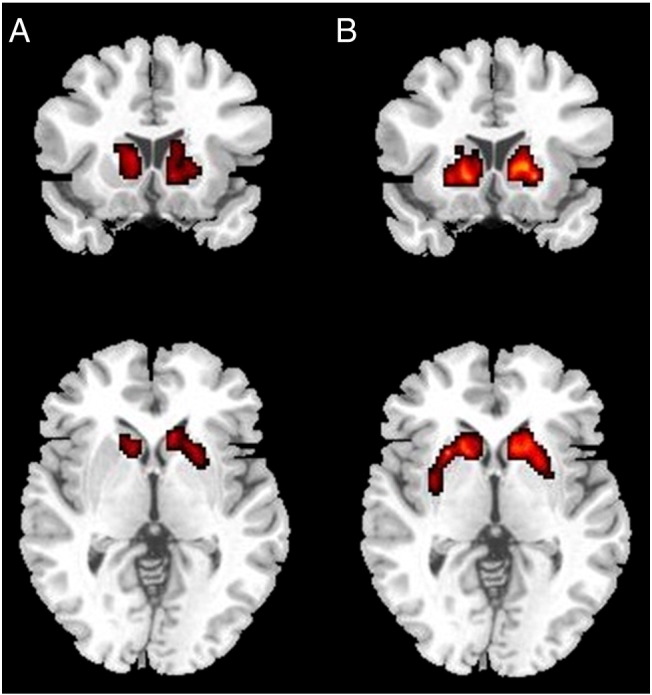
Results of voxelwise regression of [11C]-raclopride BP_ND_ in the Pathological Gamblers, showing negative association with Negative Urgency (A) (y = +15, z = − 5) and Positive Urgency (B) (y = +15, z = − 5).

The voxelwise group comparison confirmed no significant differences in [11C]-raclopride binding between PG and controls. The voxelwise regression against Negative Urgency in the PG group confirmed an inverse relationship with [11C]-raclopride binding in bilateral foci extending from the ventral putamen to the head of caudate on the right (peak co-ordinates: x = 10, y = 17, z = − 5, cluster size = 227, p < 0.001 cluster corrected) and the left caudate body (peak co-ordinates: x = − 10, y = 13, z = 2, cluster size = 103, p = 0.001 cluster corrected). The regression against Positive Urgency yielded bilateral foci extending from the nucleus accumbens and the ventral putamen through to the caudate body on the right (peak co-ordinates: x = 21, y = 15, z = − 5, cluster size = 409, p < 0.001 cluster corrected) and the left caudate and putamen (peak co-ordinates: x = − 25, y = 13, z = − 2, cluster size = 297, p < 0.001 cluster corrected) (see Fig. 3). To assess the specificity of these correlations to mood-related impulsivity, we also entered (lack of) Planning as a predictor of [11C]-raclopride binding; no supra-threshold voxels were detected.

## Discussion

We detected no differences in striatal dopamine D_2_/D_3_ receptor availability between males with PG attending a specialist treatment service, and age-matched male healthy controls. In addition to quantifying overall striatal D_2_/D_3_ receptor availability, the ROI analysis also examined receptor availability in three functional subdivisions of the striatum. The limbic subdivision comprises the nucleus accumbens, ventral putamen and ventral caudate, and is implicated extensively in addictive disorders including disordered gambling (Linnet et al., 2011; O'Sullivan et al., 2011; Steeves et al., 2009). Our observation of no differences in baseline striatal dopamine D_2_/D_3_ receptor availability between PG participants and controls is consistent with two recent [11C]-raclopride studies that assessed the change in binding as PG subjects performed different decision-making / gambling tasks (Joutsa et al., 2012; Linnet et al., 2011), and with a third PET study comparing patients with Parkinson's Disease with and without impulse control disorders including PG, during the viewing of reward-related images (O'Sullivan et al., 2011). We were unable to substantiate the reduction in BP_ND_ values reported by Steeves et al (2009) in 7 cases with Parkinson's Disease, with dopamine-agonist induced PG. It should be noted that their baseline scans involved motor selection (serial choice between four card decks with meaningless feedback), which could distort estimates of baseline availability (Egerton et al., 2009).

Several possible inferences may be drawn from our findings in PG. First, the reductions in dopamine D_2_/D_3_ receptor availability previously described in substance users (Fehr et al., 2008; Heinz et al., 2004; Martinez et al., 2004; Volkow et al., 1997, 2001) may be precipitated by the neuroadaptive or neurotoxic properties of the drugs themselves, and not aligned with the premorbid vulnerability to addictive disorders. Consistent with this account, D_2_ receptor availability was negatively correlated with the duration of stimulant abuse in a primate experimental model (Nader et al., 2006). An alternative interpretation is that reductions in dopamine binding may represent a risk factor for drug addiction that does *not* generalise to PG as a behavioural addiction. Reduced dopamine receptor availability was previously reported in obesity (Wang et al., 2001), as another candidate behavioural addiction. Of course, our findings in no way preclude the possibility of changes other neurotransmitter systems in PG, such as glutamate, GABA or serotonin (Leeman and Potenza, 2012), or indeed changes in other aspects of dopamine transmission such as release (Linnet et al., 2011), reuptake (Cilia et al., 2010) or metabolism (Bergh et al., 1997). A second inference from our findings is that any dopamine release induced by the chronic schedules of winning and losing experienced by problem gamblers (e.g. Zald et al., 2004) may not be sufficient to down-regulate striatal D_2_/D_3_ receptor availability.

Nevertheless, within the group of gamblers, [11C]-raclopride binding was negatively correlated with impulsivity, an established risk factor for problem gambling (Slutske et al., 2005) and substance use disorders (Ersche et al., 2010). The facet of impulsivity that we identify as predictive of striatal dopamine binding was Urgency (or ‘rash impulsiveness’), the tendency to commit impulsive acts under intense mood states. Case–control comparisons on the Urgency facet yielded stronger effect sizes than ‘narrow’ impulsivity (i.e. lack of planning, lack of perseverance), which was also seen in a larger group recruited from the same clinical setting (Michalczuk et al., 2011). The lack of planning subscale did not predict striatal dopamine binding in the voxelwise analysis. Urgency scores predict transitions into problem gambling, substance abuse and other risky behaviours in college samples (Cyders and Smith, 2008). Little is known about the brain systems that regulate this specific facet of impulsivity, although a recent magnetic resonance spectroscopy study reported Urgency correlations with GABA levels in dorsolateral prefrontal cortex (Boy et al., 2011), and rodent data demonstrate regulation of striatal dopamine levels by cortical GABA (Matsumoto et al., 2005). In the PG group, both negative and positive aspects of Urgency predicted striatal dopamine D_2_/D_3_ receptor availability. These relationships were seen in the overall striatal ROI as well as the limbic (Negative Urgency) and associative (both Negative and Positive Urgency) striatal subdivisions, and were confirmed in the voxelwise analysis. Negative and Positive Urgency scores were themselves inter-related, although it remains unclear whether individual patients with PG are similarly vulnerable to positive (e.g. euphoria) and negative (e.g. boredom, depression) emotional triggers (Blaszczynski and Nower, 2002; Stewart and Zack, 2008).

The relationship between Urgency and dopamine receptor availability was not manifested in the small group of healthy controls tested here. Indeed, the coefficient for Negative Urgency in the limbic striatum was significantly stronger in the PG group than the controls. In humans with methamphetamine dependence, striatal D_2_/D_3_ receptor availability was also negatively correlated with trait impulsivity (Lee et al., 2009). In light of a recent report in healthy participants that illustrated a quadratic relationship between ventral striatal [11C]-raclopride binding and trait Sensation Seeking (Gjedde et al., 2010), we performed a post-hoc analysis to test a similar effect for trait Urgency in our pooled sample of PG and healthy controls. We replicated the ‘inverted U’ (or Yerkes-Dodson) effect in the limbic striatum, as a function of Negative Urgency. This indicates that both high *and* low levels of mood-related impulsivity are associated with low striatal raclopride binding, and that BP_ND_ is maximal in the mid-range of the distribution.

In principle, lowered BP_ND_ may reflect decreases in the expression of striatal D_2_/D_3_ dopamine receptors and/or increases in synaptic dopamine levels. A study using alpha-methyl paratyrosine (AMPT) to deplete dopamine in cocaine-dependent subjects indicated reduced receptor availability coupled with *reduced* extracellular dopamine levels under baseline conditions (Martinez et al., 2009). However, preliminary work in PG has indicated increased task-related dopamine release in a subset of PG participants who reported task-related excitement (Linnet et al., 2011). It is conceivable that low BP_ND_ in low impulsives may be driven by reduced D2/D3 receptor availability, whereas low BP_ND_ in high impulsives may be driven by both reduced receptor availability and elevated extracellular dopamine (Gjedde et al., 2010). Increases in dopamine release may also be associated with compensatory down-regulation of (midbrain) autoreceptor availability, as detected with the [18F]-fallypride ligand (Buckholtz et al., 2010). In the present data, some caution is warranted by the fact that the quadratic term was driven primarily by the PG participants falling on the right-hand, descending limb, coupled with the pronounced trait difference in that group. To clarify these relationships, further studies are needed employing multiple markers of dopamine function across the full range of the impulsive trait, but we would recommend that future studies consider both linear and quadratic individual differences throughout the dopamine pathway.

As further limitations of the present study, our group sizes were small and therefore the study was not adequately powered to detect small effect sizes. Based on the effect size for the overall striatum (Cohen's *d* = 0.22), two groups of at least 350 participants would be needed to detect a statistically significant difference. Second, the use of healthy controls from a normative database imposed some minor procedural differences between the two groups: the PG subjects were presented with neutral images during their scan (albeit with no response requirement), and the PG subjects received a bolus injection whereas the controls received a bolus plus infusion. Prior work indicates that [11 C]-raclopride binding values generated by the bolus only approach are almost identical to binding values generated by a bolus-infusion approach in the same volunteers (Carson et al., 1997; Ito et al., 1998). In the PG participants, there was a degree of heterogeneity in the timing of the scan relative to treatment (most but not all were scanned pre-treatment), and psychiatric comorbidities were present in four participants. Post-hoc analyses indicated that our effects were at least not attributable to smoking status (Busto et al., 2009; c.f. Fehr et al., 2008). Psychiatric comorbidities are of course highly prevalent in PG (Kessler et al., 2008) and the inclusion of such cases does enhance the generalizability of our findings.

In terms of clinical application, it is notable that while the dopamine agonist pramipexole and partial agonist aripiprazole have been implicated in the initiation of disordered gambling (Smith et al., 2011; Voon et al., 2009), dopamine D_2_ receptor antagonists such as olanzapine have as yet failed to demonstrate overall efficacy in PG treatment trials (Fong et al., 2008; McElroy et al., 2008). Within groups of healthy participants, baseline dopamine availability influences the action of dopaminergic agents in accordance with the inverted U model (Cools and D'Esposito, 2011), and thus our findings of heterogeneity in dopamine dysregulation in disordered gambling imply that individuals with high mood-related impulsivity may show differential benefits from dopamine-based medications. For example, if low [11 C]-raclopride binding in high impulsive gamblers is attributable to increased synaptic dopamine (Gjedde et al., 2010), these individuals may be respond preferentially to dopamine blockade. The quadratic relationships with individual differences certainly imply that multiple dopaminergic mechanisms may be at work in addictive disorders (Buckholtz et al., 2010; Cilia et al., 2010; Gjedde et al., 2010).

## Disclosures

L Clark declares consultancy for Cambridge Cognition plc. AR Lingford-Hughes has received honoraria from Janssen-Cilag, Pfizer, Servier, and from the British Association for Psychopharmacology. She has provided consultancy to NET Device Corp, received research funding from Archimedes, Lundbeck, Pfizer and Schering, and holds research grants with GlaxoSmithKline. DJ Nutt has served on the advisory boards for Lundbeck, Servier, Pfizer, Reckitt Benkiser, D&A Pharma, and has also received honoraria from Bristol Myers Squibb, Glaxo Smith Kline and Schering-Plough. He has received research funding from P1vital, has share options with P1vital, and receives editorial honoraria from Sage. AR Lingford-Hughes and DJ Nutt are both members of the Lundbeck International Neuroscience Foundation. Dr Stokes, Dr Wu, Ms Michalczuk, Ms Benecke, Dr Egerton, Dr Watson, Dr Piccini and Dr Bowden-Jones have no financial interests to declare.

## Figures and Tables

**Table 1:** Striatal dopamine D2/3 receptor binding potentials and facets of impulsivity in the Pathological Gamblers and healthy Controls, with effect sizes reported as Cohen's *d*.

	PG	Controls	*d*
*Raclopride BP_ND_*			
Overall striatum	2.46 (0.26)	2.41 (0.21)	0.22
Limbic	2.22 (0.28)	2.24 (0.22)	0.08
Associative	2.38 (0.27)	2.29 (0.23)	0.35
Sensorimotor	2.78 (0.30)	2.76 (0.23)	0.11

*UPPS-P impulsivity*			
Negative Urgency	38.7 (4.33)⁎⁎	24.9 (4.32)	3.19
Positive Urgency	37.8 (6.10)⁎⁎	24.6 (6.91)	2.03
(lack of) Planning	29.3 (6.78)⁎	22.1 (6.53)	1.08
(lack of) Perseverance	22.4 (5.25)	20.8 (3.58)	0.35
Sensation seeking	36.0 (5.87)	33.1 (7.74)	0.43

⁎⁎ p < .005.

⁎ p < .05.

**Table 2:** Partial correlation co-efficients (controlling for age) in the Pathological Gamblers between [11C]-raclopride BP_ND_ in the striatal regions of interest and trait Urgency (Negative, Positive). Values in bold were statistically significant after Bonferroni correction for multiple comparisons (p = .006).

	PG		Controls	
Negative	Positive	Negative	Positive
Overall	− **0.881**	− **0.941**	− 0.298	− 0.085
Limbic	− **0.873**	− 0.857	0.193	0.311
Associative	− **0.864**	− **0.968**	− 0.365	− 0.121
Sensorimotor	− 0.724	− 0.720	− 0.099	− 0.248
